# Cavity and entrance pore development in ant plant hypocotyls

**DOI:** 10.3389/fpls.2023.1234650

**Published:** 2023-09-07

**Authors:** Hirokazu Tsukaya, Yutaka Ohtake

**Affiliations:** ^1^Graduate School of Science, The University of Tokyo, Tokyo, Japan; ^2^Graduate School of Engineering, The University of Tokyo, Tokyo, Japan

**Keywords:** *Hydonophytum*, Rubiaceae, 3D imaging, ant plant, cavity, computed tomography, gravity

## Abstract

Some genera of Rubiaceae in South-eastern Asia are known as typical ant plants. They have large domatia, which form in well-developed hypocotyls in which ants nest. Previously, cavity formation processes were described; however, these reports were dependent on tissue sections of different individuals of different ages. No continuous time-course analyses were done because cavity formation occurs inside the thick tissues of highly swollen domatia. Here we observed cavity formation processes in ant plants by using X-ray computed tomography (CT) imaging and revealed previously overlooked features of cavity formation. Firstly, the cavity pore occurs at the hypocotyl base in not only gravity-dependent but also basal position-dependent manner. Secondly, the cavity forms prior to the start of short tunnel formation between the cavity and the pore. The cavity axis is parallel to the longitudinal axis of the hypocotyl; however, the short tunnel axis between the pore and cavity depends on gravity. Non-invasive CT scanning is a very powerful method to analyze deeply hidden morphogenic processes in organs.

## 1 Introduction

Ant plants from many families have a symbiotic relationship with ants, whereby special plant organs with cavities are used as ant nests (Beattie, 1985). Rubiaceae ant plants, such as the genera *Anthorrhiza*, *Hydnophytum*, *Myrmecodia*, *Myrmephytum*, and *Squamellaria* are the most well-known ant plants that have been studied since the 1970s (Huxley, 1978; Huxley and Jebb, 1991a; Huxley and Jebb, 1991b). Common features of these genera are their domatia (highly developed hypocotyls; sometimes called tubers), cavity formation inside of the domatia, and no ant nest formation outside of hypocotyls (Huxley, 1978; Jebb, 1991). Domatia is not a simple swelling of hypocotyls. Radishes or even *Arabidopsis thaliana* hypocotyls swell after germination, but no cavity differentiates inside tissue. In contrast, ant plant domatia develop highly differentiated cavities with several types of surface walls (Jebb, 1991). Morphological and developmental studies on these Rubiaceae ant plants peaked with the memorable publication by Huxley (1978), while ecophysiological studies on their mutualism with ants are still actively conducted (Chomicki and Renner, 2016; Chomicki and Renner, 2019). Non-invasive X-ray computerized tomography (CT) imaging exhibits its power in analyzing developmental processes of complex or hidden 3D structures. Thus, CT imaging has recently been applied in plant developmental biology (Furuya et al., 2019; Sarath et al., 2019; Piovesan et al., 2021). This imaging technique has been used on Rubiaceae ant plant domatia (Chomicki and Renner, 2019); however, to the best of our knowledge, it has not been used for analyzing cavity formation. As described in some literatures (Jebb, 1991; Chomicki and Renner, 2017), cavity structure and distribution in mature domatia are quite diversified and complicated, and occur inside of thick tissue. Therefore detailed developmental processes of the cavity were not well understood. Even for the initial steps of the first cavity formation, it is also the case.

In the past one of the authors (HT) got an opportunity to observe many young seedlings of *Myrmecodia* in native habitats of Papua. They diagonally attach on tree trunks and the initial pore always opens on the downwards or lower side of the hypocotyl base, suggesting that the cavity opening position may depend on gravity (Tsukaya, unpublished observations: **Supplemental Figure 1**). Moreover, at the opening stage of the pore, the cavity appears to be already complete. This indicates that cavity formation precedes the opening of the pore. To the best of our knowledge, these processes have not been described in detail.

The development of the domatia from a thin hypocotyl was already described 140 years ago in sketches of several ant plants at different developmental stages (Treub, 1883). In most of these cases, the first pore was formed in the basal part of the hypocotyl; thereby supporting our idea that pore formation is influenced by gravity. However, no physiological studies regarding this have been done. Moreover, in past review articles it was stated that “tuber cavities are made by phellogens, meristematic layers which arise *de novo* in the parenchyma of the swollen hypocotyl and enclose volumes of parenchyma” and “following suberization of a single layer of cell walls, the enclosed tissue dies and shrivels” (Huxley and Jebb, 1991a). In this scenario, cavity formation and pore (entrance hole) opening is a single-step event. Huxley (1978) stated that dead cells are “normally removed by the ants”. Moreover, it was reported that “the first cavity is initially a simple hook shape in all the genera, except some members of *Myrmecodia* where the apex is bifid”. However, no time-course analyses were conducted to confirm this. All the above issues are due to difficulties in observing the processes of cavity formation, because all these processes take place inside thick tissues. Therefore, this study analyzed the processes of cavity formation in the young stages of seedlings under cultivation. Here we use CT imaging to describe the processes of the very early stages of the first cavity formation and pore opening in some Rubiaceae ant plants.

## 2 Results and discussion

### 2.1 Initial steps of domatia formation

**Figure 1:**
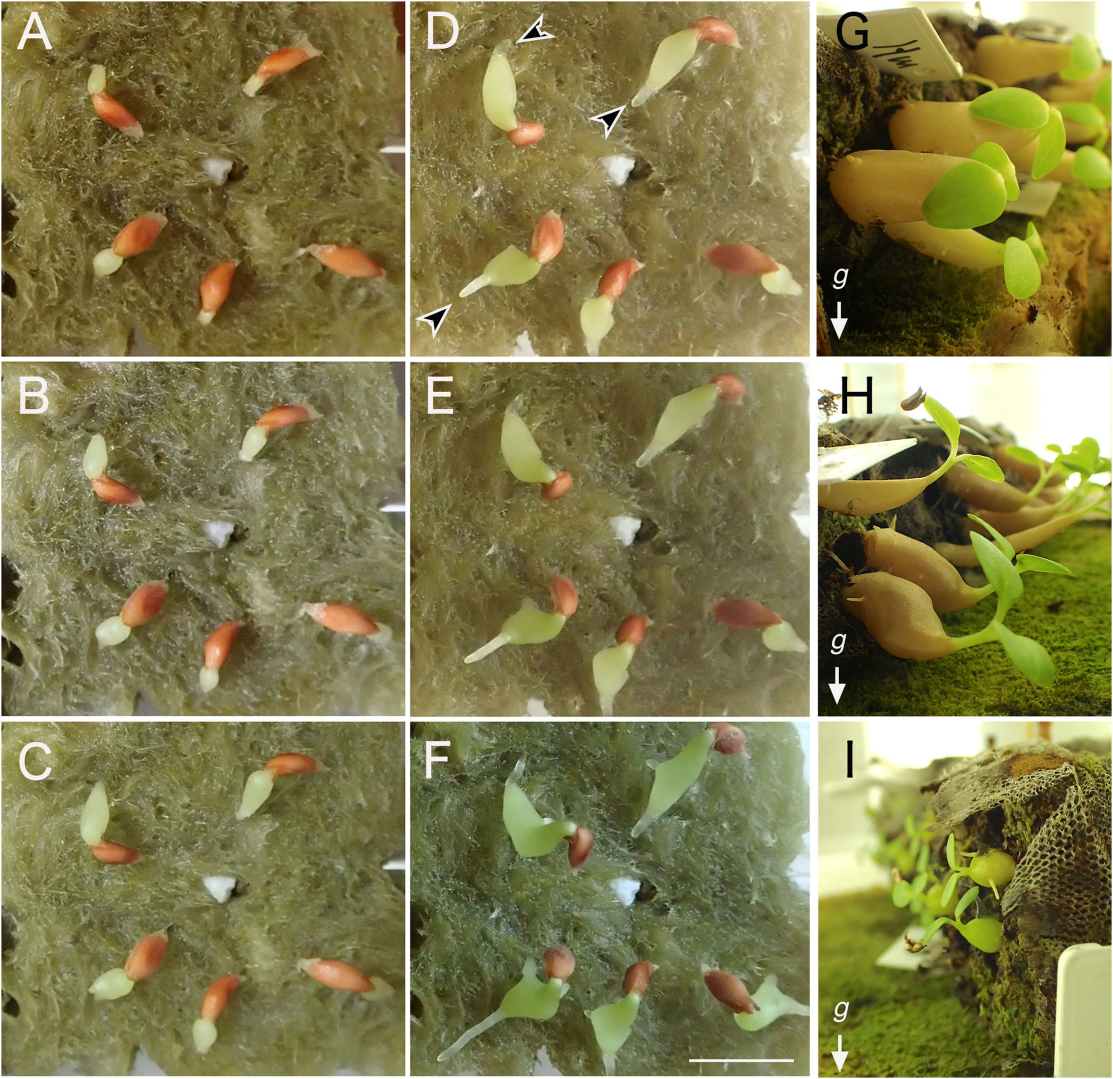
Development of domatia after germination. Early steps of seedling development in *Hydonophytum moseleyanum* on rockwool **(A)** 1 day, **(B)** 2 days, **(C)** 3 days, **(D)** 5 days, **(E)** 6 days, and **(F)** 8 days after sowing. Arrowheads indicate primary development of hypocotyl before emergence/elongation of root in panel **(D, E)** Cotyledon opening did not occur even after significant thickening of hypocotyl and elongation of roots. Scale = 1 cm. Approximately 2-month-old seedlings of **(G)** *(H) moseleyanum*, **(H)** *Myrmecodia tuberosa*, and **(I)** *Myrmephytum beccarii*. Note gravitropism is absent in the hypocotyl but expressed in stems.

Firstly, early seedling development was monitored for four ant plant species: *Anthorrhiza bracteosa* C.R. Huxley & Jebb, *Hydnophytum moseleyanum* Becc., *Myrmecodia tuberosa* Jack, and *Myrmephytum beccarii* Elmer. The early steps of seedling development after germination were similar among the species. First, a thick hypocotyl appeared from the seed (**Figure 1A**) and an extensive hypocotyl thickening occurred without elongation of the main root (**Figures 1B–E**). Main and adventitious root elongation followed before extension of cotyledons (**Figure 1F**). Extension of cotyledons occurred 20-25 days after sowing. Similarly, unlike usual angiosperm seedlings, the Rubiaceae ant plant seedlings paused for a long time after cotyledon expansion for shoot apical meristem activity (**Figures 1G–I**). The first pair of foliage leaves become to be enough large to be seen by naked eye more than three months after sowing. Until it they therefore only developed a hypocotyl to make domatia (**Figure 1**) as described by Huxley (1978), assimilates obtained from expanded cotyledons are mostly consumed for development of hypocotyls. It is known that development of sink capacity of hypocotyls in radish is dependent on an ability of sucrose storage governed by sucrose synthase (Usuda et al., 1999). It is plausible that the very strong sink capacity in the swelling hypocotyl/domatium in Rubiaceae ant plants might be also regulated by such sucrose storage ability. Physiological examination from this view point is a future issue.

Interestingly, negative gravitropism was not recognized in the thickened part of hypocotyls, whereas it was observed in the thin, apical part of hypocotyls (**Figure 1H**). After the formation of the pore, we observed that the cavity was already established, suggesting that cavity formation precedes the opening of the pore.

### 2.2 Dependency on gravity

As mentioned in the Introduction, our past observations of pore formation in the ant plants’ native habitat urged us to examine whether gravity indeed affected the position of the pore. As a preliminary test, *Myrmecodia tuberosa* seedlings with their natural positions were examined for the pore position seven months after sowing. All examined seedlings had pores at the basal part of the hypocotyl; 23 seedlings had the pore on the lower side whereas eight seedlings had it on the lateral side. Therefore, it is strongly suggested that pore position is determined by two factors: positional cue (basal position of the hypocotyl) and gravity.

To examine the influence of gravity, two weeks after the germination the seedlings of four species were laid sideways by fixing the position of the Jiffy-7 base to be tilted at a 90-degree angle (**Table 1**; **Figures 1G–I**). Due to the limited number of available seeds, other conditions for gravity stimulus timing were not tested. After 14-22 weeks, the position of the pore was checked. As shown in **Table 1**, all seedlings formed pores at the base of the hypocotyl, indicating that the potential for pore formation is restricted to the basal part of the hypocotyl at the seedling stage. Moreover, almost all seedlings (see **Table 1**) showed pore formation on the lower side of the hypocotyl basal region, clearly indicating that the pore formation site is also dependent of gravity. While the bilateral symmetry axis made by the pair of cotyledons was also considered for the positioning of pores in *Hydnophytum moseleyanum*, no correlation was recognized between the plane of the cotyledons and the pore position (three individuals made pore parallel to the cotyledon plane and three made perpendicular to the cotyledon plane). Therefore, pore site determination was concluded to be developmentally (at the base of hypocotyl) and environmentally (by direction of gravity) controlled.

**Table 1:** Position of the pore in reference to gravity.

Species	Pore opening position (number of individuals)			
	Lower side^a^	Lower lateral	Upper side^a^	n.d.^b^
*Anthorrhiza bracteosa*^d^	7	0	0	5
*Hydnophytum moseleyanum*^e^	5	1 ^c^	0	0
*Myrmecodia tuberosa* ^e^	8	3 ^c^	1	0
*Myrmephytum beccarii* ^d^	10	0	0	0

All pores differentiated at the base of hypocotyls. We determined the pore location for which, lower (bottom) or upper (top).

^a^ bottom or top side ± 15 degree, respectively.

^b^ not differentiated yet at the observation.

^c^ formed at near lateral side beyond 15 degree from the bottom.

^d^ Observed 24 weeks after sowing.

^e^ Observed 16 weeks after sowing.

This conclusion is reasonable considering the ecological aspects of the domatia. In the native habitat in Papua, nearly all seedlings were attached on the surface or cracks of tree trunks in a diagonal manner (**Supplemental Figure 1**). In this orientation, if the pore formed in the upper side of the domatia, raindrops would penetrate into the cavity and cause severe damage for tissues under construction of cavities *via* some infections of harmful fungi and bacteria. Therefore, the pore location on the loser side is beneficial to ant plants.

### 2.3 Variations in the cavity

**Figure 2:**
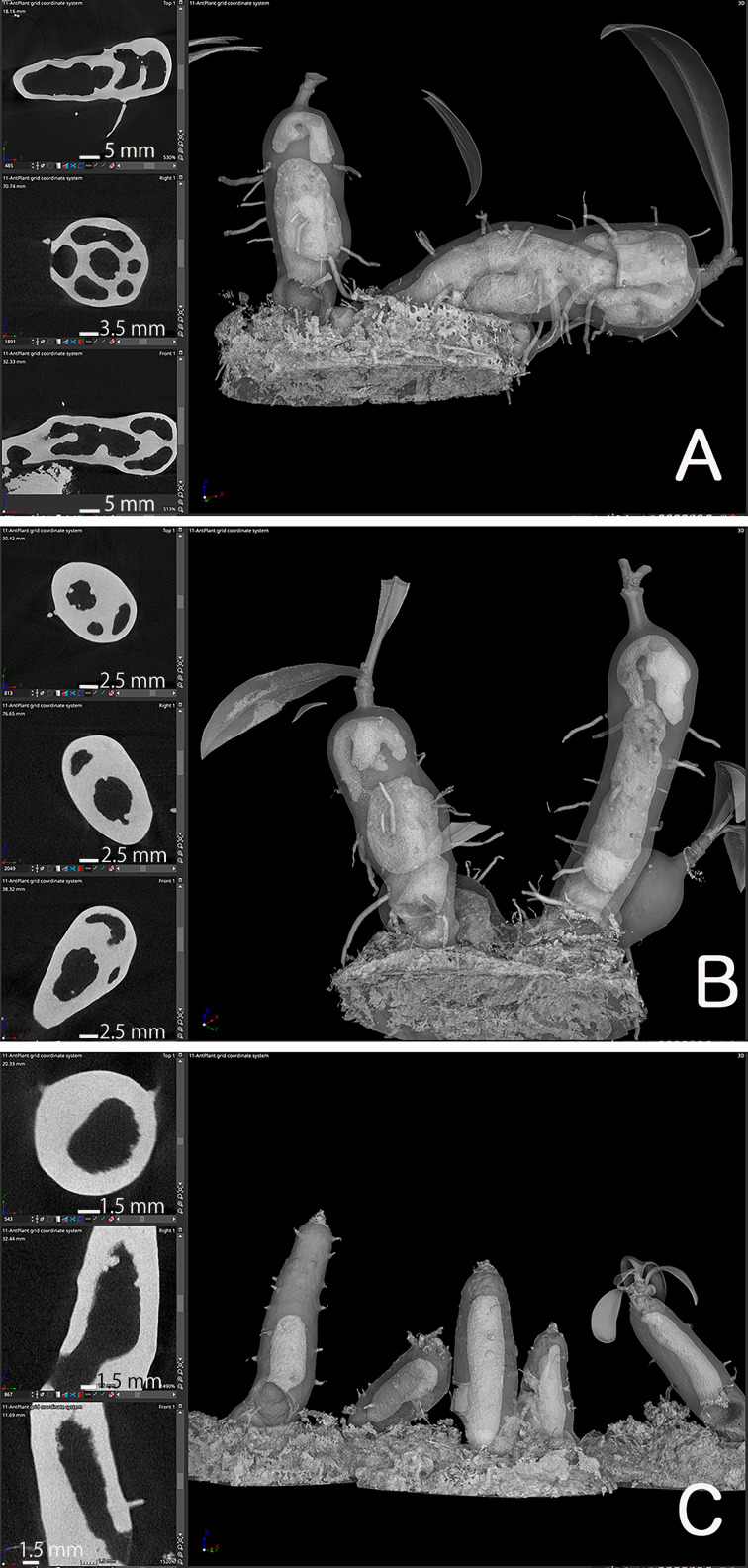
Cavity shape variations. Micro computed tomography images of **(A, B)** *Myrmecodia tuberosa* and *Hydonophytum moseleyanum* seedlings at 7.5 months after sowing. In each panel, the left three images are X-, Y-, and Z-sectional view (top to bottom) reconstructed as described in Materials and Methods and the right image is gross morphology with 60% transparency. Note the **(A, B)** branched and irregular cavity shape of *M. tuberosa* compared with **(C)** single and large cavity of (*H*) *moseleyanum*.

Once the location of the pore was determined, we investigated how the cavity was formed using micro CT observations. Preliminary examination showed that domatia tissue was easily observed without any staining or pre-treatment; thus, we observed the cavity of species from two major genera with past reports on their cavity shape. The *Myrmecodia* and *Hydnophytum* species had the earliest pore formation among the four species examined (**Table 1**), and the initial cavity shape differed between them. At 7.5 months after sowing, *M. tuberosa* showed very complex, branched cavities in the hypocotyl whereas *H. moseleyanum* had a single, large cavity (**Figure 2**). This is similar to past reports by Huxley (1978) which state that “the shape of the first cavity does not change markedly except in some *Myrmecodia* spp”. However, Huxley (1978) described the *Myrmecodia* cavity apex as bifid, which differs from the present observations of a branching pattern (**Figure 2**). In many cases, the first cavity connected to the pore had an upside-down U-shape (**Figures 2A, B**). In some cases, the upside-down U-shaped cavity had short or long extensions at the apex, forming a Y-shaped cavity (**Figures 2A, B**). Therefore, it appears that the upside-down U-shape is a basic form of the first internal cavity in *M. tuberosa.* Recognition of such variation is a benefit of the CT observations that was not easy in the past due to the difficulty of section-based studies to grasp the 3D shape of the cavity.

In addition, a unique character was also recognized for the *M. tuberosa* internal cavity. As seen in **Figures 2A, B**, an additional and independent cavity was formed at the apical region of the hypocotyl in *M. tuberosa.* The apical cavity was found to be isolated from the basal cavity which was connected by a pore and had a complicated looped shape with branching. This should be a secondary cavity described before (*e.g.*, Jebb, 1991). In the past the role and fate of the first cavity in the mature plants was in a debate. Future time-course studies of CT-based cavities will fix the matters.

### 2.4 Processes of cavity formation

We focused on the *Hydonophytum* species cavity formation, because this species consistently formed the same single, large cavity in the hypocotyls. From serial observations we found several new features of the cavity that were not previously described.

**Figure 3:**
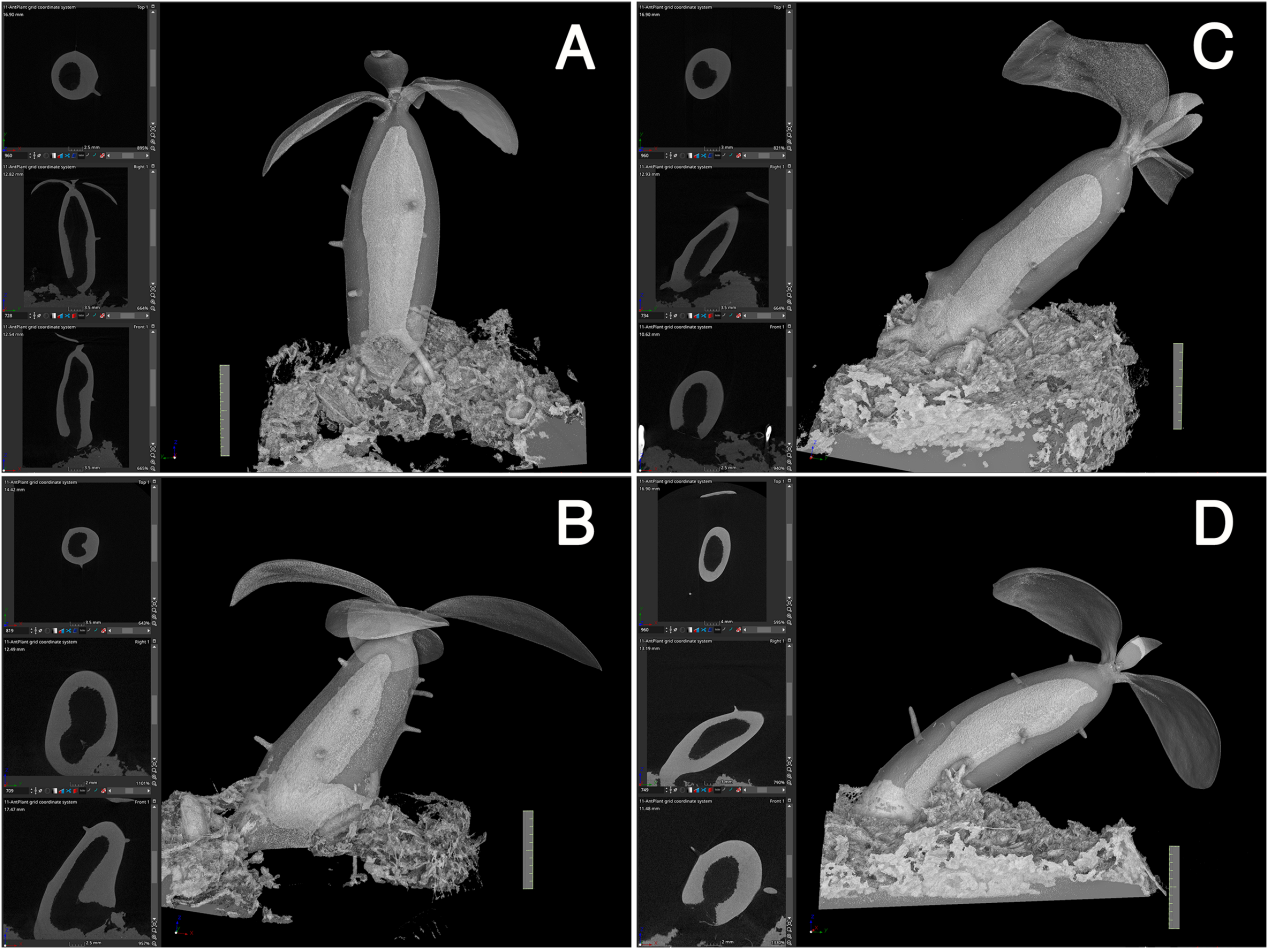
Cavity is parallel to the longitudinal axis of the hypocotyl. **(A–D)** Four representative individuals of *Hydonophytum* seedlings 11 weeks after sowing. X-, Y- and Z- cross sectional images for each, gross 3D image rendered to be 50-60% transparency, are shown. Note the straight shape of the cavity formed parallel to the longitudinal axis of the hypocotyl, irrespective of the orientation of plantlets against gravity (shown by the direction of the scale with 1 cm). A short tunnel connecting the cavity and pore is, however, directionally distinct from the cavity.

Firstly, the cavity shape was always straight. Among the 14 seedlings examined, all the cavities were straight and parallel to the longitudinal axis of the hypocotyl, independent of the seedling orientation, and irrespective of whether the hypocotyls stood straight or obliquely (**Figure 3**). This observation indicated that the cavity shape is determined innately and is independent of gravity. The cavity extended to the apical and basal regions of the hypocotyl and ended apically beneath the shoot apex, remaining several millimeters away (**Figure 3**).

**Figure 4:**
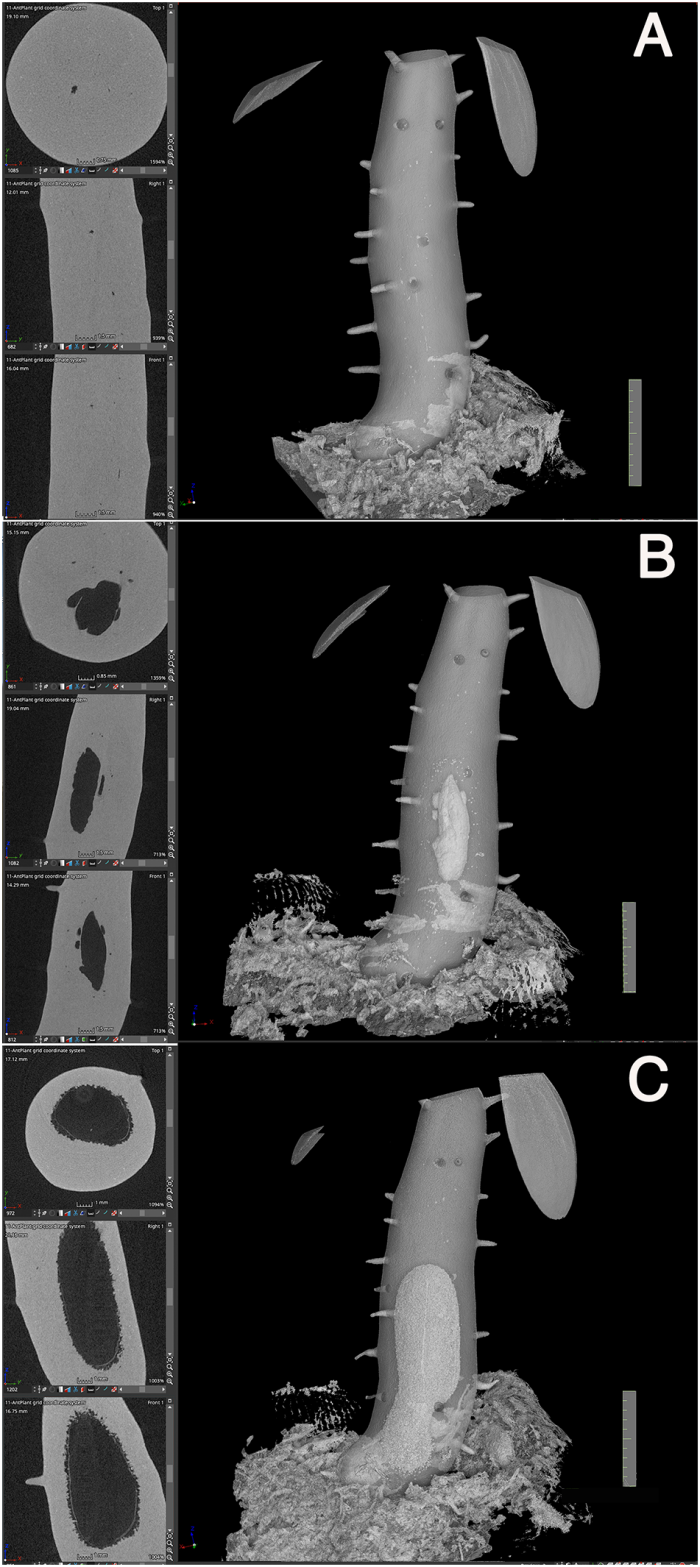
Cavity formation. Three successive processes of cavity formation are shown for a representative *Hydonophytom* seedling. **(A)** 11 weeks, **(B)** 13 weeks, and **(C)** 15 weeks after sowing. X-, Y- and Z- cross sectional images are shown for each gross 3D image rendered to be 50-60% transparency (left panel; top to bottom). Note expansion of the internal cavity that started from the centre of the hypocotyl, and the connection of small pore or sponge formation into a large cavity. Scale = 1 cm.

Secondly, initiation of the cavity started from the center region in terms of the diameter and from the mid region of the hypocotyl in terms of the longitudinal axis (**Figure 4**). Initially, small punctate pores (**Figure 4A**), which appeared to be a symptom of cell death, were formed and made the tissue sponge-like. Subsequently, they appeared to fuse into a large cavity (**Figure 4B**). Surrounding tissues of the cavity were then occupied with continuous formation of rough spongy structures that were then fused into the central cavity. The cavity formation expanded longitudinally and a single, large, and straight cavity was made (**Figure 4C**).

**Figure 5:**
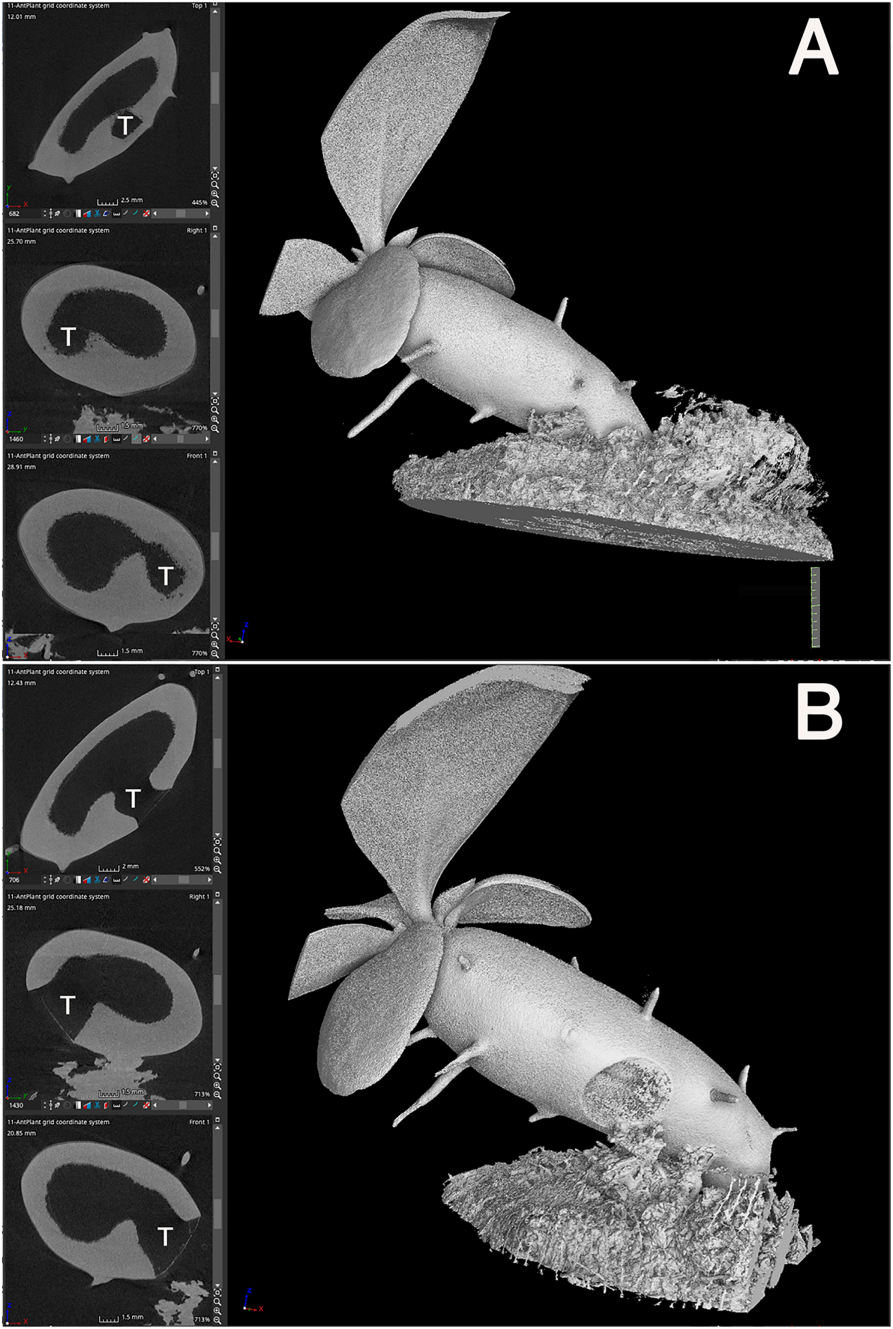
Short-tunnel formation. A representative *Hydnophytum* seedling of **(A)** 11 weeks and **(B)** 13 weeks after sowing., X-, Y- and Z- cross sectional images are shown for each gross surface 3D image (left panel; top to bottom). **(A)** A short tunnel (shown by T) connecting the cavity with the pore started to form from the inner side, **(B)** and then extended to form a pore. Note that the direction of the cavity axis and the short-tunnel axis is nearly perpendicularly crossed. **(B)**The remaining thin dead epidermis is also seen at the pore. Scale = 1 cm.

Thirdly, pore formation did not occur until the above-mentioned inside first cavity reached to the bottom (**Figure 4C**). This is contradictory to the past understanding that of cavity and pore forming in a single step after determination of the phellogens-surrounding region (Huxley, 1978). Instead, it was found that pore formation only started after cavity formation was completed. As shown in **Figures 3** and **5**, the cavity is always straight and parallel to the longitudinal axis of the hypocotyl, and the short tunnel that connects the basal most end of the cavity and the pore starts to form from the inner side (**Figure 5A**). The short tunnel then extends to the surface of the hypocotyl to open the pore (**Figure 5B**). Considering the fact that the inside cavity formation starts from the mid region and extends in both apical and basal directions, this unidirectional extension of the short tunnel appears to be another unique feature. The common feature between the cavity and the short tunnel for the pore is that they both arise from degradation of sponge-like tissue (**Figures 4**, **5**). In some cases, the pore was covered with thin tissue (peel), (**Figure 5B**) but often this structure had naturally fallen off after shrinkage.

**Figure 6:**
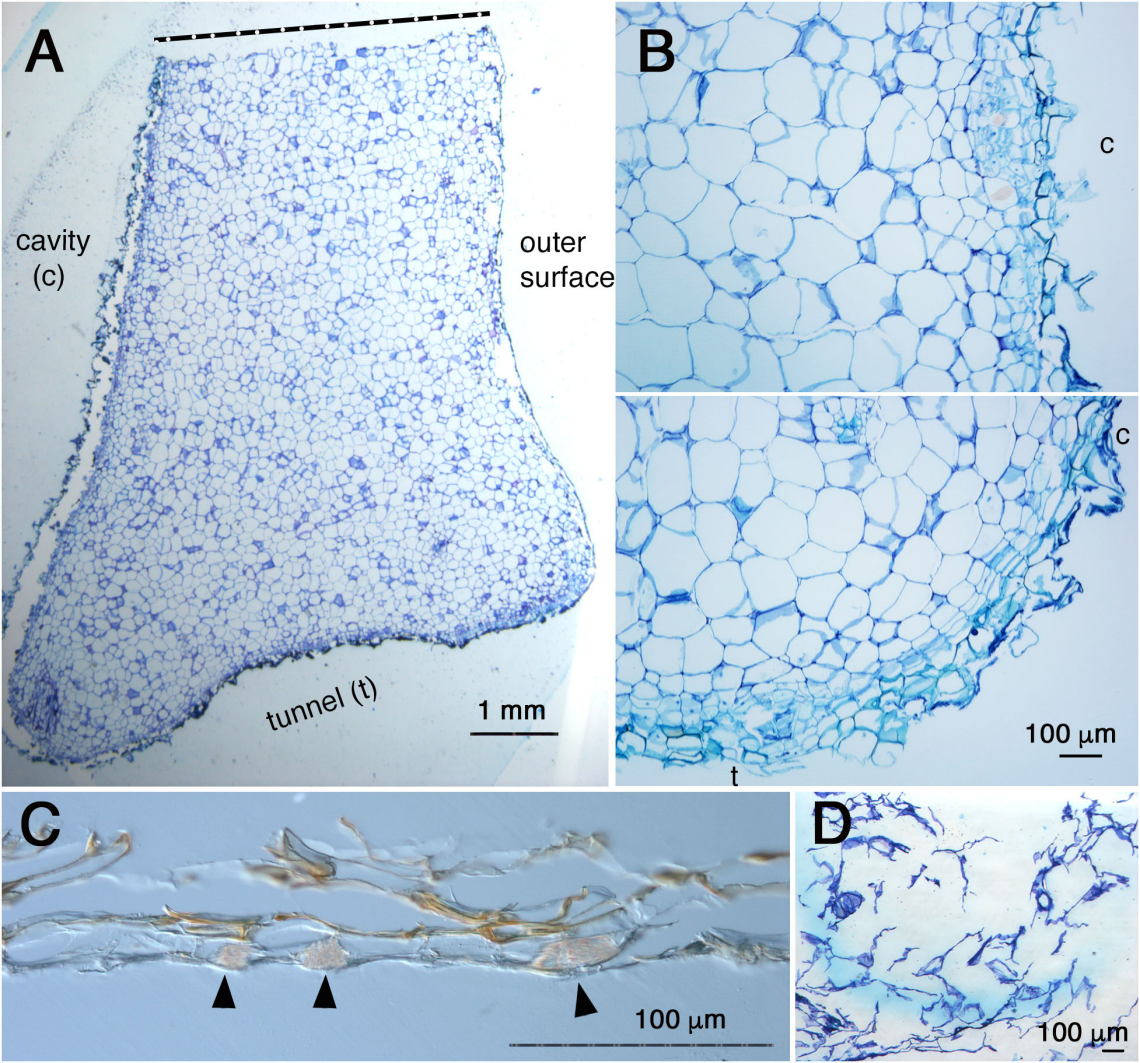
Anatomy of cavity and pore ‘peel’ surfaces. **(A)**, Micrograph of a section around the pore and cavity of *Hydnophytum.* Upper side is a cut side for sampling; bottom side is a surface of a short tunnel connecting the pore and cavity; left side is the wall surface that surrounds the cavity; and right side is the outer hypocotyl surface. Scale = 1 mm. **(B)**, Magnified view of the surface of the cavity (above) and a corner between the cavity (c) and the short tunnel (t) to the pore (bottom). Note the similarity of the tissue structure and surface covered with thin dead cells between the cavity and the short tunnel surfaces. Scale = 100 μm. **(C)**, Cross section of the ‘peel’ of a pore (no staining with toluidine blue). Note the multicellular layers and brown pigmentation. Idioblasts with crystals of calcium oxalate are shown by blac arrowheads. Scale = 100 μm. **(D)**, Attached ‘peel’ debris. Sponge-like dead cells with low density are seen. Scale = 100 μm.

Fourthly, the inside cavity became completely empty even before pore formation as shown in **Figures 2**, **4** and **5**. This observation indicates that the dead cells simply thrived. This is also inconsistent with the past statement that inner dead cells are “normally removed by the ants” (Huxley, 1978).? To elucidate how these dead cells were removed, the complete cavity region including the peel of the pore was cross-sectioned (**Figure 6**). As a result, it was found that the surface of the cavity and the short tunnel are both covered with thin, dead, multicellular layers (**Figures 6A, B**). This was also true for the peel of the pore (**Figure 6C**), indicating that dead cells were reduced to very thin tissue. Still in an exceptional case, a small fragment of white, sponge-like tissue remained attached to the peel and was found to be a low-density cluster of dead cells (**Figure 6D**). Therefore, majority of dead cells are dried up and lost in the space, but some remaining dead tissues might be removed by ants in nature. Clear cell files were not seen in majority of the parenchymatous tissue of the domatia (**Figure 6A**), except on the surface where two to three cell files were recognized parallel to the surface layer (**Figure 6B**). In some cases, crystals of calcium oxalate, that are also seen in parenchymatous tissue in living tissue as idioblasts, were seen among the dead cells in the peel (**Figure 6D**).

### 2.5 CT-based 3D serial observation is effective in analyzing cavity formation in ant plants

The present study revealed many hidden processes of cavity formation in ant plants that differ from past descriptions. The inconsistency appears to be derived from the methodology used for observations. In the past, time-course analysis was impossible because all the processes, except for the final step of peel formation and its removal, occur inside of thick tissue. Moreover, previous studies on cavity formation made comparisons of sections from different individuals of different ages; therefore, there was room for speculation regarding the connection between the different samples. However, as revealed in this study, all the steps of the first cavity formation is done deep inside tissue and no symptom is observed from outside until the pore opening. In addition, most past studies were carried out by using a limited number of wild individuals; therefore, it was difficult to capture each developmental step in high resolution.

In this study non-invasive CT scanning was used to solve the above difficulties in combination with cultivation under controlled conditions without association with ants. As a result, many hidden characters in the process of cavity formation could be revealed. Recently, the use of CT imaging has expanded in Plant Science; however, its use is limited (Piovesan et al., 2021). The present study demonstrates a powerful application of CT for analyzing plant morphogenesis, and it provides a rationale for further use of CT imaging by plant scientists. Importantly, no staining or pre-treatment was required for visualization of the cavities in this study, thereby enabling us to do time-course observations without damaging the seedlings.

Moreover, it was revealed that the pore position is dependent on gravity. This finding was also achieved by cultivation of seedlings under controlled conditions without ants. To date, many developmental processes have been shown to be affected by gravity response in plants (Nakamura et al., 2019); however, a suggestion of gravity-dependent positioning of the pore for symbiosis with ants is a new. Such gravity responses are often mediated by auxin signaling, which is also a major signaling pathway for controlling organ formation and morphogenesis in plants (Vanneste and Friml, 2009; Swarup and Bhosale, 2019). Moreover, in many cases auxin works as a positional cue (Teale et al., 2006; Hellmann, 2021). In plants, the most studied auxin-regulated autonomous cavity formation is aerenchyma formation in water-submerged plants such as rice (Colmer, 2003; Yamaguchi et al., 2019). For example in rice roots where extensive studies have done for mechanisms of aerenchyma formation, it was revealed that a part of *AUXIN/INDOLE-3-ACETIC ACID PROTEINS* (*AUX/IAA*) genes (*IAA13*) and a member of *AUXIN RESPONSE FACTOR* (*ARF*) gene (*ARF19*) are involved in aerenchyma formation co-acting with a LATERAL BOPUNDARY DOMAIN (LBD)-CONTAINING PROTEIN (LBD1-8) (Yamaguchi et al., 2019). Treatment with an auxin polar transport inhibitor, N-1-naphthylphthalamoc acid, was shown to decrease the aerenchyma formation in rice root that could be partially rescued by application of IAA. Thus, future physiological studies can focus on whether the gravity response, in terms of pore positioning, is mediated by auxin signaling.

## 3 Materials and methods

### 3.1 Plant materials

Fresh seeds of Rubiaceae ant plants were obtained from STRINGEPLANTS (Nagoya, Aichi, Japan). Seeds were sown on Jiffy-7 (Sakata Seed Corporation, Yokohama, Japan) fixed on rockwool (Toyobo) moistened with tap water. Cultivation was done at 23°C, under continuous illumination with light-emitting diode or white fluorescent light at 40-60 μmoles m^-2^ s^-1^ as described by Tsuge et al. (1996). To examine the influence of gravity direction for the pore location determination, soon after extension of cotyledons, we changed the position of seedlings. Namely, seedlings were laid sideways by fixing the position of the Jiffy-7 base on rockwool to be tilted at a 90-degree angle (**Figures 1G–I**).

### 3.2 Tissue sectioning

Sectioning, staining and microscopic observations were carried out as described by Tsuge et al. (1996). Briefly, tissue samples were fixed with formalin-acetic acid-alcohol solution and dehydrated with ethanol series and embedded in Technovit 7100 resin (Kulzer and Co., Wehrheim, Germany), followed by 10-12 μm-thickness sectioning with a microtome (Microm HM360, Thermo Fischer Scientific, USA). Sections were stained with 0.1% (w/v) toluidine blue (Sigma) in 0.1 M phosphate buffer (pH 7.0), washed with water, dried, and mounted on glass slides with Entellan new mounting medium (EMD, Millipore, Burlington, MA, USA). Microphotography was done using a differential interference contrast microscope (DM4500, Leica, Germany).

### 3.3 CT imaging

CT imaging was done without any staining or pre-treatment of plant individuals cultivated on Jiffy-7 under 130 kV, 300 μA, and high resolution scan mode using a micro-CT scanner (Rigaku CT Lab HX; Rigaku, Japan). The voxel size of 3D-CT image was 37.4 μm. The 3D-CT images were analyzed in iso-surface rendering without applying any image filters. The obtained series of images were integrated into 3D images from where x-, y-, and z- sections were automatically reconstructed using myVGL software (Volume Graphics Co., Ltd., Nagoya, Japan).

## Data availability statement

The original contributions presented in the study are included in the article/**Supplementary Material**. Further inquiries can be directed to the corresponding author.

## Author contributions

HT designed and carried out the analyses, YO conducted the micro-CT operations. HT and YO wrote the manuscript. All authors contributed to the article and approved the submitted version.
