# Supplementary material for: Cavity and entrance pore development in ant plant hypocotyls
Source: Front Plant Sci. 2023 Sep 7;14:1234650. doi: 10.3389/fpls.2023.1234650 (PMC10513446; doi:10.3389/fpls.2023.1234650)
Supplement: Supplementary file 1 [file DataSheet_1.pdf]

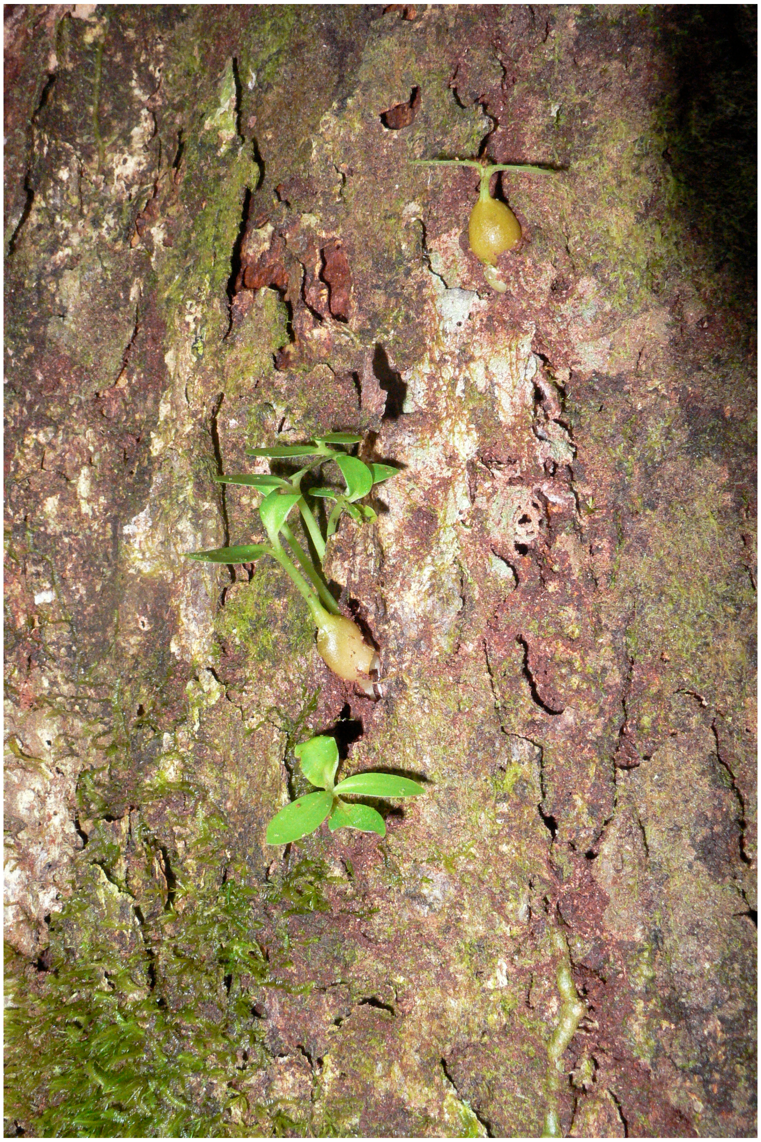

Supplementary Figure 1. *Myrmecodia beccarii* seedlings observed in native habitat, Papua in 2008

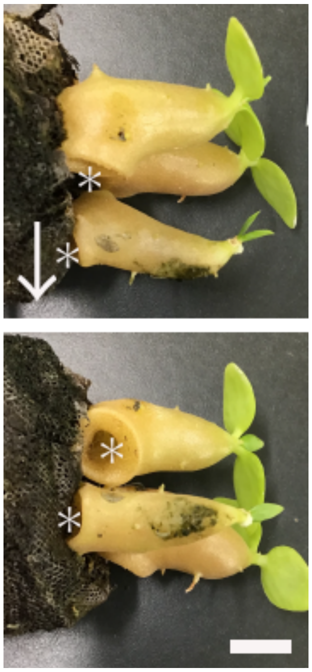

Supplementary Figure 2. Pore opening position in *Hydonophytum moseleyanum*

A white arrow indicates the direction of gravity. Star marks indicate location of the pores. Upper panel, lateral view; lower panel, bottom view. Scale indicates 1 cm.
